# Integrated mRNA and miRNA Analysis Reveals Layer-Specific Mechanisms of Antler Yield Variation in Sika Deer

**DOI:** 10.3390/ani15131964

**Published:** 2025-07-04

**Authors:** Derui Zhao, Zhen Zhang, Qianghui Wang, Heping Li

**Affiliations:** 1 College of Wildlife and Protected Area, Northeast Forestry University, Harbin 150040, China; zhaoderui@nefu.edu.cn (D.Z.); wangqianghui@nefu.edu.cn (Q.W.); 2 Institute of Animal Husbandry and Veterinary, Heilongjiang Academy of Agricultural Reclamation Sciences, Harbin 150036, China; dongnongzz@126.com

**Keywords:** sika deer, antler growth, transcriptomics, miRNA-mRNA network

## Abstract

Antlers exhibit exceptionally rapid growth, representing a rare biological phenomenon among mammals. In addition to their scientific significance, antlers are widely used in traditional medicine, and their yield directly impacts the economic efficiency of the deer farming industry. However, antler yield varies substantially among individuals, and the molecular mechanisms underlying this variation remain poorly understood. This study aimed to elucidate the transcriptomic and post-transcriptional mechanisms underlying antler yield variation by comparing gene and miRNA expression profiles across four distinct antler tissue layers—dermis (D), reserve mesenchyme (RM), pre-cartilage (PC), and cartilage (C)—in sika deer with different yields. RNA-seq and miRNA-seq were performed, followed by differential expression, GO and KEGG pathway enrichment, and miRNA–mRNA co-expression network analyses. Our results reveal layer-specific expression patterns and key regulatory genes and miRNAs associated with proliferation, chondrogenesis, angiogenesis, and mineralization. In particular, genes such as *FBP2*, *TPT1*, *TFRC*, *ZEB1*, and *PHOSPHO1* were upregulated in high-yield deer across specific tissue layers, while *NFATC2* was downregulated in these high-yield deer. Additionally, miRNAs such as miR-140, miR-296-3p, and let-7e exhibited layer-specific expression patterns linked to growth and differentiation. Our miRNA–mRNA regulatory network analysis highlighted significant interactions, particularly miR-296-3p–PHOSPHO1 and miR-296-3p–FBP2, as key regulators of antler growth. Enrichment of PI3K-Akt and TGF-β signaling pathways further suggests their involvement in promoting chondrogenesis and ossification. These findings provide novel insights into the molecular basis of antler growth and yield, which may inform future strategies for selective breeding in deer farming.

## Simple Summary

Deer antler, a traditional Chinese medicine rich in amino acids, vitamins, and minerals, is also an important health product that is popularly used in Asian countries. However, antler yields vary widely among individuals, affecting the economic efficiency of the deer farming industry. In this study, we analyzed different tissue layers of growing antlers from high- and low-yield sika deer using RNA and microRNA sequencing technologies. We found that candidate genes and microRNAs are differentially expressed across tissue layers in individuals with different antler yields. These findings enhance our understanding of the molecular mechanisms underlying antler growth and yield, and may contribute to the development of selective breeding strategies to improve antler production in farmed deer.

## 1. Introduction

Antler, the unossified and pilose antler of male sika deer (*Cervus nippon*) or red deer (*Cervus elaphus*), represents the only fully regenerable and rapidly growing organ in mammals, making it an excellent model for studying rapid tissue growth and skeletal development [1,2]. In traditional Chinese medicine, antler has been widely used as a therapeutic agent and is associated with various pharmacological effects, including immunomodulatory [3], anti-inflammatory [4], anti-fatigue [5], anti-osteoporotic [6], neuroprotective [7], and anti-tumor effects [8]. Due to its biological and medicinal importance, sika deer are widely domesticated in East Asia. In Asian countries, such as the Republic of Korea, China, and Mongolia, antlers have been traditionally used for thousands of years [9]. The annual global production of velvet antler is currently estimated at approximately 1300 tons and continues to grow rapidly to meet increasing demand in the medicinal market [10]. However, there exists substantial individual variation in antler yield, commonly measured by antler weight. Antler growth is a complex process influenced by genetic, physiological, and environmental factors. Elucidating the molecular mechanisms underlying this variation is critical for improving breeding strategies and enhancing antler production.

**Figure 1:**
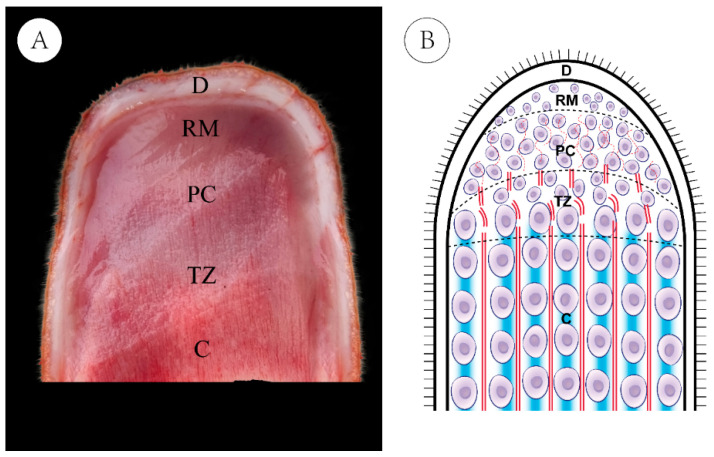
Longitudinal section of the antler growth center (AGC). (**A**) Histological section of the AGC. D: Dermis; RM: Reserve mesenchyme; PC: Pre-cartilage; TZ: Transition zone; C: Cartilage. (**B**) Schematic diagram of the AGC, showing the cellular arrangement and vascular distribution across different tissue layers.

During the period of antler rapid growth, antlers can reach growth rates of up to 2.75 cm per day [11], which primarily occurs at the tip and is driven by rapid proliferation and differentiation of cells within the antler growth center (AGC) [12]. Histologically, the antler tip is composed of five distinct layers: the dermis (D), reserve mesenchyme (RM), pre-cartilage (PC), transition zone (TZ), and cartilage (C) (Figure 1A). These layers exhibit unique morphological and functional characteristics (Figure 1B). The D layer contains a richly vascularized, innervated skin that provides protection and metabolic support. The RM layer harbors undifferentiated mesenchymal cells, serving as a reservoir for proliferative and chondrogenic precursors. The PC layer consists of early chondrogenic cells and initial vascular structures, marking the onset of cartilage formation. The TZ layer, located between the PC and C layers, histologically presents as a mixed tissue exhibiting distinct features derived from both. The TZ layer is generally recognized as a transitional stage in the developmental progression from PC layer to C layer. The C layer contains mature chondrocytes and established vasculature, contributing directly to longitudinal growth and subsequent ossification [13,14,15].

Advances in high-throughput RNA-seq have significantly enhanced our understanding of gene expression dynamics during antler development [16,17]. RNA-seq has enabled the identification of differentially expressed genes (DEGs), transcriptional regulators, and signaling pathways involved in AGC function. Notably, pathways such as Wnt signaling have been implicated in stem cell activation, chondrogenesis, and osteogenesis [18]. Additionally, unique modifications in tumor suppressor genes may contribute to rapid tissue proliferation in antler growth [19]. Comparative transcriptomic studies across AGC layers have identified transcription factors, growth factors, and extracellular matrix components associated with rapid tissue growth [20,21]. Furthermore, microRNAs (miRNAs) have been shown to play critical roles in post-transcriptional regulation within antler cartilage, particularly in pathways related to metabolism and cancer [22,23].

Recent studies have made significant progress in elucidating the genetic basis of antler yield in sika deer. Whole-genome resequencing studies have identified numerous genetic variations associated with antler weight. For instance, Recent research identified 94 genetic variants linked to this trait, revealing genetic diversity and differentiation among populations [24]. Furthermore, genome-wide single nucleotide polymorphism (SNP) analysis has shown that farmed sika deer populations possess relatively low genetic diversity, which may impact key productive traits such as velvet antler yield [25]. Comprehensive transcriptome analyses have revealed the dynamic expression profiles of crucial genes during antler development, including COL2A1 and SOX9, which play pivotal roles in chondrogenesis and ossification [26]. Moreover, high-quality genome sequencing has identified key transcription factors, such as RUNX2 and members of the SOX family, that regulate the processes of antler growth and ossification [27]. Despite substantial progress in understanding the molecular mechanisms underlying antler growth, significant gaps remain. Notably, differences in transcriptomic profiles between high- and low-yield sika deer across the RM, PC, C, and D layers have yet to be systematically characterized. These comparative analyses offer valuable insights into the regulatory mechanisms underlying differential growth potential, thereby informing selective breeding strategies in deer farming.

In this study, we performed a comprehensive transcriptomic analysis of the AGC tissue layers in high- and low-yield sika deer. By comparing gene expression profiles across RM, PC, C, and D layers, we aimed to uncover molecular mechanisms underlying differential antler growth and yield. Our findings provide new insights into the genetic regulation of rapid tissue proliferation and differentiation, contributing to advancements in both agricultural and biomedical applications.

## 2. Materials and Methods

### 2.1. Ethics Statement and Sample Preparation

All animal procedures and handling procedures were approved by the Institutional Animal Care and Use Committee of Northeast Forestry University (2025066).

Six healthy 4-year-old male sika deer were selected and raised under identical conditions. Based on antler weight, they were divided into high-yield (H) and low-yield (L) groups, with group differences in antler weight analyzed by *t*-test, revealing a statistically significant difference between groups (*p* < 0.01) (Table 1). Samples were collected 75 days after the shedding of previous hard antlers. The growing antler tips (7–9 cm) were excised, longitudinally sectioned along the sagittal axis, and immediately dissected into four tissue layers: dermis (D), reserve mesenchyme (RM), pre-cartilage (PC), and cartilage (C). Each layer was cut into 4–6 mm pieces, flash-frozen in liquid nitrogen, and stored at −80 °C for RNA extraction and sequencing.

### 2.2. RNA-Seq Sequencing and Bioinformatics Analysis

Total RNA was extracted using the Trizol reagent kit (Invitrogen, Carlsbad, CA, USA) following the manufacturer’s instructions. RNA quality was assessed using an Agilent 2100 Bioanalyzer (Agilent Technologies, Palo Alto, CA, USA) and verified by RNase-free agarose gel electrophoresis. Eukaryotic mRNA was enriched from the total RNA using Oligo (dT) beads, then fragmented into short fragments using fragmentation buffer. The fragmented mRNA was reverse-transcribed into cDNA using the NEBNext Ultra RNA Library Prep Kit for Illumina (NEB#7530, New England Biolabs, Ipswich, MA, USA). The resulting double-stranded cDNA was purified, end-repaired, A-tailed, and ligated to Illumina sequencing adapters. The ligation products were purified using AMPure XP beads (1.0×), size-selected via agarose gel electrophoresis, and amplified by polymerase chain reaction (PCR).

The resulting cDNA libraries were sequenced using the Illumina X Ten platform. The raw sequencing data included reads containing adapters and low-quality bases, which can affect downstream assembly and analysis. Therefore, high-quality clean reads were obtained by filtering the raw data using fastp (version 0.18.0). An index of the reference genome was constructed, and the paired-end clean reads were aligned to the reference genome (GCA_040085125.1) using HISAT2 (version 2.2.4). Mapped reads for each sample were assembled using StringTie (version 1.3.1) in a reference-guided manner. Read counts for each gene were calculated using HTSeq-count (version 0.9.1) with the “union” mode and “gene_id” as the feature identifier, based on the corresponding GTF annotation file. Gene expression levels were quantified as fragments per kilobase of transcript per million mapped reads (FPKM). Differential expression analysis was performed using DESeq2 between the two groups. Genes with a false discovery rate (FDR) < 0.05 and an absolute |log2foldchange| ≥ 1 were defined as differentially expressed genes (DEGs).

### 2.3. miRNA-Seq Sequencing and Bioinformatics Analysis

Total RNA was extracted using the Trizol reagent kit (Invitrogen, Carlsbad, CA, USA), and small RNA molecules ranging from 18 to 30 nucleotides were enriched by polyacrylamide gel electrophoresis (PAGE). After ligation of 3′ adapters, RNA fragments of 36–44 nucleotides were selected, followed by 5′ adapter ligation, reverse transcription, and PCR amplification. PCR products of 140–160 bp were purified to construct a cDNA library, which was subsequently sequenced on the Illumina NovaSeq 6000 platform. Raw sequencing reads were subjected to quality control using the software fastp (version 0.18.0) to remove adapter sequences, low-quality reads, and reads with ambiguous nucleotides, thereby generating high-quality clean data. These clean data were first aligned to the Rfam database (Release 11.0) to remove non-miRNA small RNAs, including rRNA, scRNA, snoRNA, snRNA, and tRNA. Remaining tags were mapped to the reference genome, and those aligning to exonic or intronic regions—likely originating from degraded mRNA—as well as reads mapped to repetitive sequences, were excluded. Known miRNAs were identified by aligning the clean reads to the miRBase database (Release 22), while unannotated tags were further mapped to the reference genome and assessed for novel miRNA prediction using miRDeep2, based on genomic location and predicted hairpin structures. Both known and novel miRNAs constituted the total miRNA dataset. Expression levels were normalized to transcripts per million (TPM), and differential expression analysis was performed using DESeq2. miRNAs with |log2foldchange| ≥ 0 and *p*-value < 0.05 were considered significantly differentially expressed.

### 2.4. Analysis of GO and KEGG Pathway

Gene ontology (GO) is a standardized system for functional gene annotation that provides a continuously updated controlled vocabulary to describe gene products in terms of their associated biological processes, cellular components, and molecular functions. GO enrichment analysis identifies GO terms that are significantly overrepresented among differentially expressed genes (DEGs) compared with the genomic background, thereby highlighting the biological functions potentially involved. In this study, DEGs were mapped to GO terms using the Gene Ontology database “http://www.geneontology.org/ (19 February 2025)”, and the number of genes assigned to each term was calculated. Significantly enriched GO terms were identified using a hypergeometric test. To further investigate the biological significance of the DEGs, pathway enrichment analysis was conducted based on the Kyoto Encyclopedia of Genes and Genomes (KEGG) database https://www.kegg.jp (19 February 2025). As genes typically function in coordinated pathways, KEGG enrichment analysis enables the identification of significantly enriched metabolic or signaling pathways associated with the DEGs relative to the whole-genome background.

### 2.5. Construction of miRNA-Target Gene Co-Expression Network

In our study, four software programs, RNAhybrid (v2.1.2), svm_light (v6.01), Miranda (v3.3a), TargetScan (Version: 7.0), were used to predict miRNA targets. miRNA sequences and family information were obtained from TargetScan website http://www.targetscan.org/ (6 March 2025). We evaluated the expression correlation between differentially expressed miRNAs and their predicted target genes using the Pearson correlation coefficient (PCC). Pairs with PCC < −0.7 and *p* < 0.05 were selected as negatively co-expressed miRNA-target pairs. The miRNA-target gene network was constructed as above, and then visualized using Cytoscape software (v3.6.0) http://www.cytoscape.org/ (10 April 2025).

### 2.6. Validation of DEGs by qRT-PCR

To validate the reliability of our transcriptome data, we selected 8 DEGs for quantification by quantitative real-time reverse transcription PCR (qRT-PCR). Total RNA was extracted from tissue samples using the Animal RNA Extraction Kit (Aikore, AG21024) and its concentration and purity were determined with a Multiskan Sky microplate spectrophotometer (Thermo Scientific, Waltham, MA, USA). First-strand cDNA synthesis was carried out using the TransScript^®^ RT/RI Enzyme Mix (Genscript, Singapore, AE311-02), and the resulting cDNA was stored at −80 °C until further use. qRT-PCR amplifications were performed on an H-9800 instrument (Hehui Bio, Suzhou, China) with M5 HiPer SYBR^®^ Premix Es Taq™ (MF015; Polymei, Beijing, China). Reactions were assembled according to the manufacturer’s protocol, and relative gene expression levels were calculated using the 2^−∆∆CT^ method, with β-actin serving as the internal reference. All qRT-PCR reactions were performed with three biological replicates per group. The difference in data was analyzed by *t* test. *p* > 0.05 was not significant, and *p* < 0.05 was significant. Primer sequences for the eight target DEGs and β-actin are presented in Table 2.

## 3. Results

### 3.1. Descriptive Statistics of mRNA Expression Analysis

**Figure 2:**
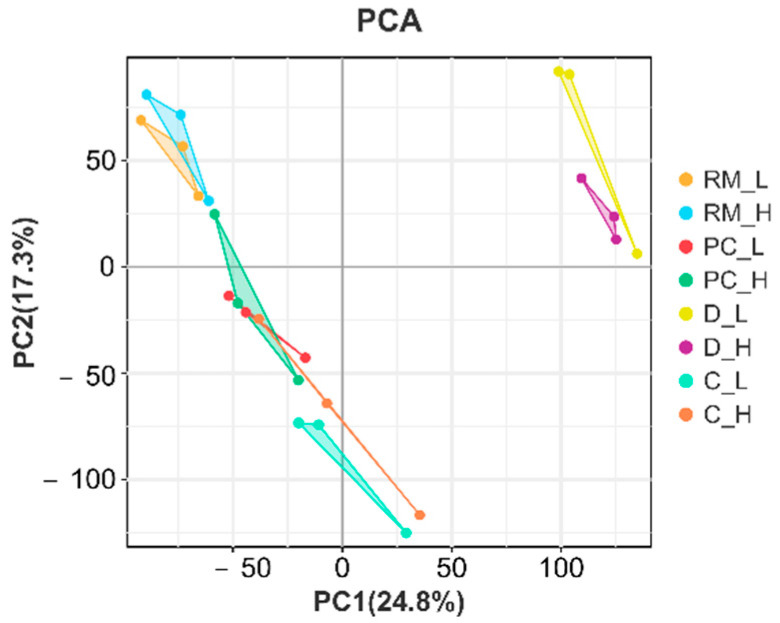
Principal component analysis (PCA) plot of transcripts identified in four tissue layers of antlers with high and low yield. The analysis revealed distinct clustering patterns among the different layers—dermis (D), reserve mesenchyme (RM), pre-cartilage (PC), and cartilage (C)—between the high-yield (H) and low-yield (L) groups, indicating layer-specific gene expression differences associated with antler yield variation.

**Figure 3:**
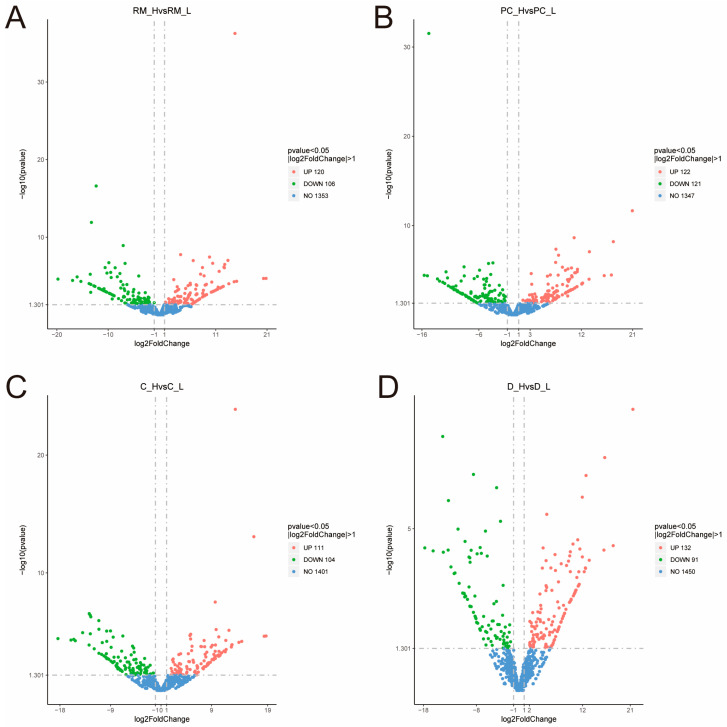
Differentially expressed genes (DEGs) between high-yield (H) and low-yield (L) groups across four tissue layers. Each point represents a gene, with the *x*-axis indicating the log2 fold change (H vs. L) and the *y*-axis indicating the statistical significance (–log10 *p*-value). Green points represent genes with significantly higher expression in the low-yield group, and red points represent genes with significantly higher expression in the high-yield group (adjusted *p*-value < 0.05 and |log2FoldChange| > 1). Blue points represent genes with no significant expression difference. (**A**) Reserve mesenchyme (RM); (**B**) pre-cartilage (PC); (**C**) cartilage (C); (**D**) dermis (D).

**Figure 4:**
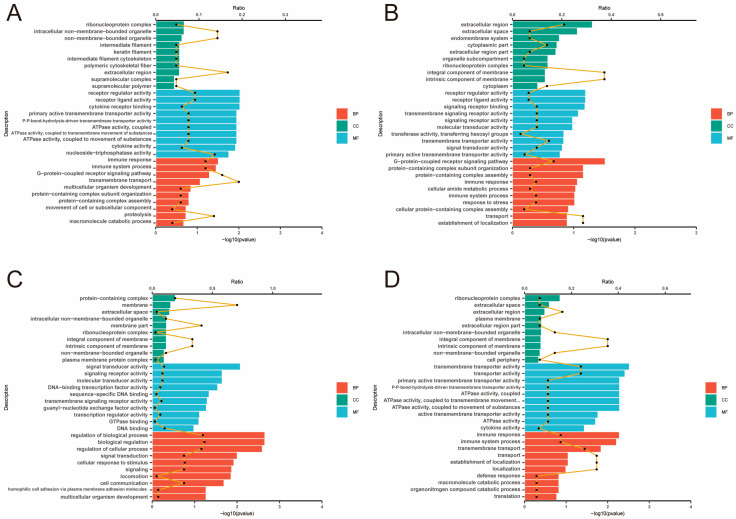
Gene ontology (GO) enrichment analysis of differentially expressed genes (DEGs) between high-yield and low-yield antlers across four tissue layers. (**A**–**D**) represent the GO enrichment analysis for DEGs (high yield vs. low yield) in the four different tissue layers of antlers: (**A**) dermis (D), (**B**) reserve mesenchyme (RM), (**C**) pre-cartilage (PC), and (**D**) cartilage (C). Enriched GO terms are categorized into three domains: biological process (BP, red), cellular component (CC, green), and molecular function (MF, blue). The yellow line graph indicates the ratio of DEGs annotated to each GO term relative to the total number of genes in that category.

**Figure 5:**
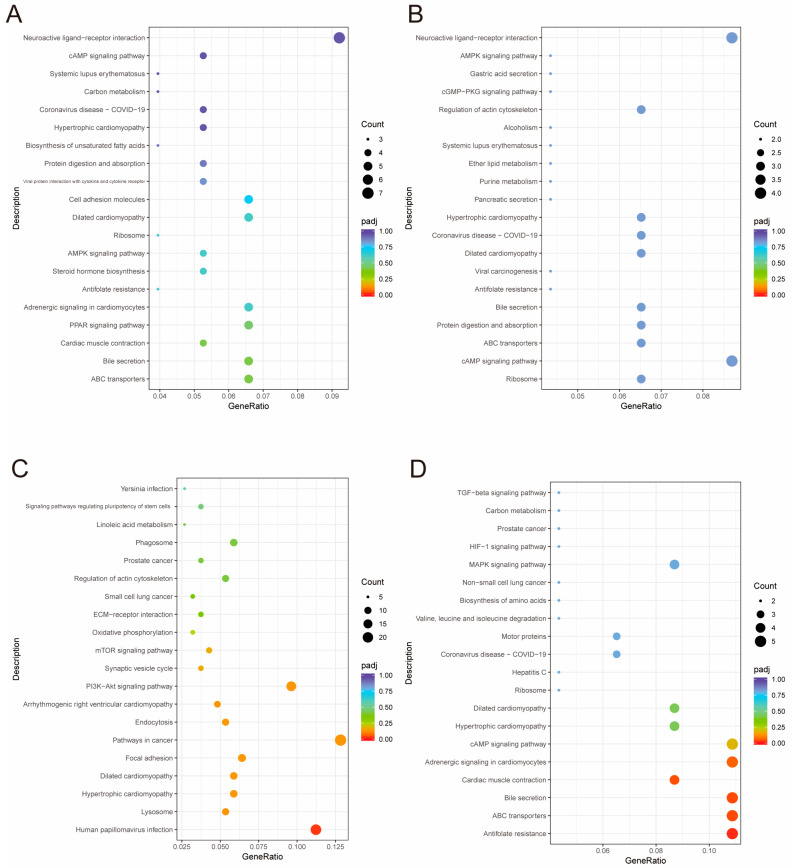
KEGG pathway enrichment analysis of differentially expressed genes (DEGs) in four different layers of high- and low-yield antlers. (**A**–**D**) The enriched KEGG pathways for DEGs (high yield vs. low yield) in the following tissue layers of antlers: (**A**) Dermis (D), (**B**) reserve mesenchyme (RM), (**C**) pre-cartilage (PC), and (**D**) cartilage (C). The x-axis indicates the gene ratio (number of DEGs involved in a pathway divided by total DEGs), the size of the dots corresponds to the number of DEGs and the color represents the adjusted *p*-value (padj). Pathways are ranked by significance and gene ratio to highlight biological processes that differ between high- and low-yield antler tissues.

Paired-end 150 bp RNA-Seq sequencing was performed using the Illumina X-Ten platform. A total of 310.02 Gb of clean data were generated from the RNA sequencing. On average, each sample yielded approximately 13.09 Gb of clean reads, with a base quality score (Q30) of no less than 93.03%. The GC content ranged from 51.13% to 52.13% across all samples. Furthermore, more than 95.5% of the transcripts from each sample were successfully mapped to the reference genome. All samples demonstrated genome alignment rates exceeding 93%, underscoring the overall high mapping efficiency and the reliability of the sequencing results (Table S1). Gene expression profiles for each replicate were evaluated using principal component analysis (PCA) (Figure 2). Differential expression analysis with DESeq2 identified a total of 223, 226, 243, and 215 DEGs in the four comparative groups, respectively (Figure 3) (Tables S2–S5). GO enrichment analysis was conducted to explore the functional roles of DEGs across different tissue layers. In the D layer (Figure 4A), DEGs were mainly enriched in biological processes such as macromolecule catabolic processes, defense responses, ATPase activity, and membrane-associated components, reflecting roles in metabolic regulation, immune defense, and energy transduction. In the RM layer (Figure 4B), DEGs were primarily associated with G-protein-coupled receptor signaling pathways, suggesting involvement in stem cell regulation. In the PC layer (Figure 4C), enriched GO terms included multicellular organism development, cell communication, and transcriptional regulation, indicating important roles in early chondrogenesis. In the C layer (Figure 4D), DEGs were enriched in stress responses, immune-related processes, and protein complex assembly, suggesting active participation in cartilage maturation and ossification. KEGG pathway analysis revealed the top 20 significantly enriched pathways in each tissue comparison group (Figure 5A–D), including the ABC transporters, AMPK signaling pathway, cAMP signaling pathway, TGF-β signaling pathway, PI3K−Akt signaling pathway, and ECM−receptor interaction. Based on the results of transcriptomic analysis, six DEGs potentially associated with antler growth were identified as candidate genes: *FBP2*, *TPT1*, *TFRC*, *NFATC2*, *ZEB1*, and *PHOSPHO1*. These were among the most significantly differentially expressed molecules between the high- and low-yield groups. Based on previous literature reports, these candidate genes may play a fundamental role in the developmental function of antlers.

### 3.2. Descriptive Statistics of miRNA Expression Analysis

**Figure 6:**
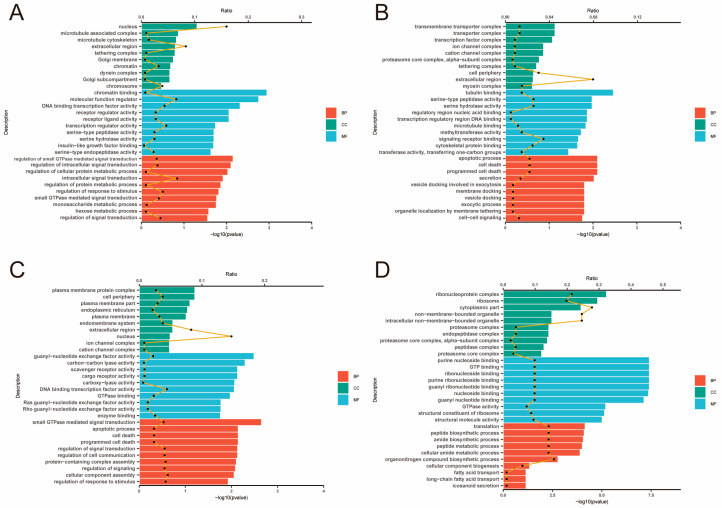
Gene ontology (GO) enrichment analysis of differentially expressed miRNA between high-yield and low-yield antlers across four tissue layers. (**A**–**D**) The GO enrichment analysis for differentially expressed miRNA (high yield vs. low yield) in the four different tissue layers of antlers: (**A**) dermis (D), (**B**) reserve mesenchyme (RM), (**C**) pre-cartilage (PC), and (**D**) cartilage (C). Enriched GO terms are categorized into three domains: biological process (BP, red), cellular component (CC, green), and molecular function (MF, blue). The yellow line indicates the proportion of differentially expressed miRNAs associated with each GO term.

**Figure 7:**
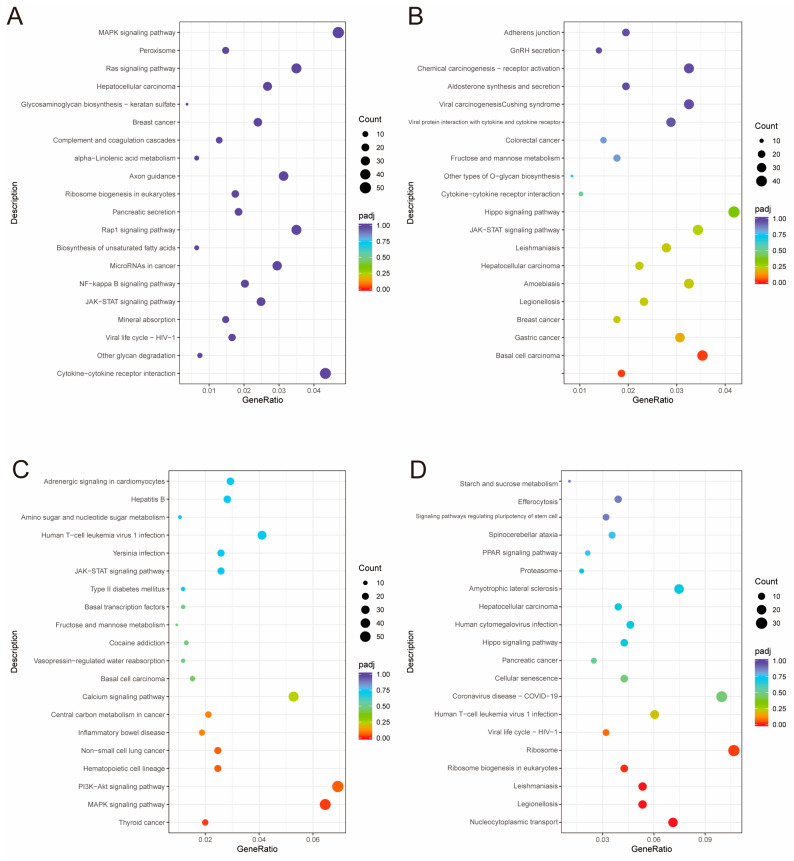
KEGG pathway enrichment analysis of differentially expressed miRNA in four different layers of high- and low-yield antlers. (**A**–**D**) The enriched KEGG pathways for differentially expressed miRNA (high yield vs. low yield) in the following tissue layers of antlers: (**A**) Dermis (D), (**B**) reserve mesenchyme (RM), (**C**) pre-cartilage (PC) and (**D**) cartilage (C). The *x*-axis indicates the miRNA ratio (number of differentially expressed miRNAs involved in a pathway divided by the total number of differentially expressed miRNAs), the size of the dots corresponds to the number of miRNAs, and the color represents the adjusted *p*-value (padj).

Single-end 50 bp miRNA sequencing was conducted using the Illumina HiSeq 6000 platform. Differential expression analysis using DESeq2 identified a total of 43, 27, 10, and 9 differentially expressed miRNAs (DE-miRNAs) in the four comparative groups (Figure S1) (Tables S6–S9), respectively. Gene ontology (GO) analysis was performed to elucidate the functional roles of the target genes of these DE-miRNAs (Figure 6). In the D layer (Figure 6A), GO terms were significantly enriched for signal transduction regulation, intracellular signaling, and metabolic processes, suggesting that DE-miRNAs play active roles in modulating cell communication and energy metabolism. In the RM layer (Figure 6B), enriched GO categories included cell–cell signaling, signaling receptor binding, transcription regulatory region DNA binding, and cell death, indicating that miRNAs in this layer may be involved in regulating intercellular communication and transcriptional activity. In the PC layer (Figure 6C), GO enrichment was observed in pathways related to signal transduction and small GTPase-mediated signaling, suggesting miRNA-mediated regulation of cell signaling and differentiation. In the C layer (Figure 6D), DE-miRNAs were mainly associated with genes involved in translation, peptide biosynthesis, and fatty acid transport, reflecting increased protein production and metabolic support for terminal chondrocyte maturation. KEGG pathway analysis revealed the top 20 significantly enriched pathways in each comparative group (Figure 7A–D), with many DE-miRNAs participating in key signaling pathways such as JAK–STAT, MAPK, Hippo, and PI3K–Akt. Based on the miRNA sequencing data, three differentially expressed miRNAs, namely miR-140, miR-296-3p and let-7e, were found to be not only significantly differentially expressed but also to play essential roles in cell proliferation and cartilage differentiation, processes closely related to antler regeneration and yield.

### 3.3. Results of Network Analysis of miRNA-mRNA

**Figure 8:**
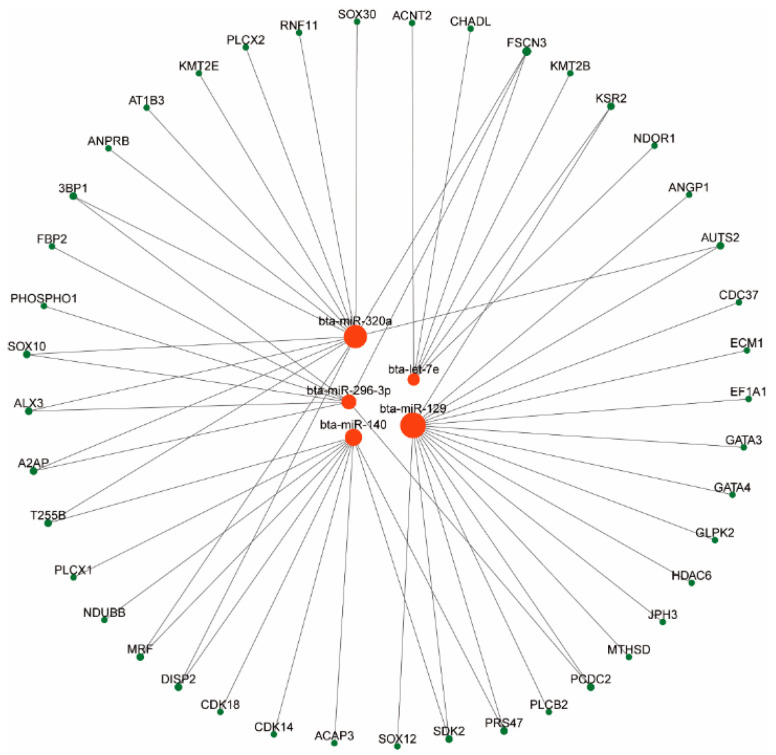
miRNA-target gene network analysis of differentially expressed miRNAs in high- and low-yield sika deer antlers.

**Figure 9:**
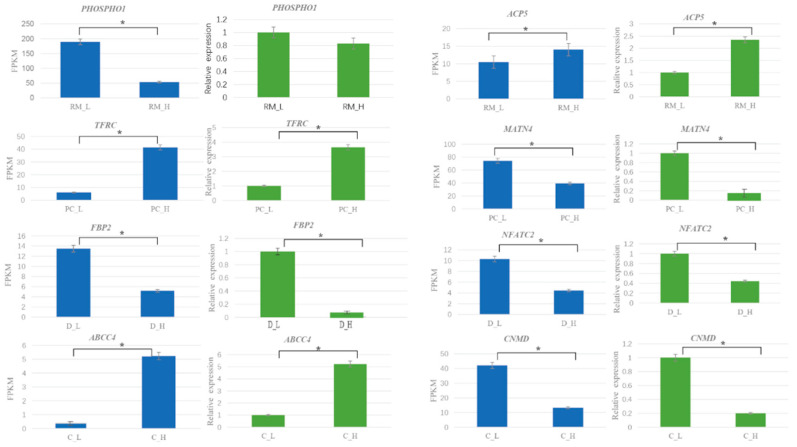
Validation of mRNA expression levels using qRT-PCR. Bar graphs show the expression levels of eight selected differentially expressed genes (DEGs)—*PHOSPHO1*, *ACP5*, *TFRC*, *MATN4*, *FBP2*, *NFATC2*, *ABCC4*, and *CNMD*—in high-yield (H) and low-yield (L) sika deer across four antler tissue layers: reserve mesenchyme (RM), pre-cartilage (PC), dermis (D), and cartilage (C). Blue bars represent transcript levels quantified by RNA-Seq and expressed as fragments per kilobase of transcript per million mapped reads (FPKM). Green bars show corresponding relative gene expression levels determined by qRT-PCR, normalized to β-actin. Error bars indicate standard deviation, and values are averaged over three biological replicates. * Indicates statistically significant differences (*p* < 0.05). Each gene displays yield-specific and layer-specific expression patterns. The expression trends between qRT-PCR and RNA-Seq data are generally consistent, validating the reliability of the transcriptomic analysis.

To elucidate key regulatory interactions involved in antler growth in sika deer, we conducted a network analysis of differentially expressed miRNAs and their predicted target mRNAs. The miRNA–mRNA interaction network was visualized using Cytoscape (Figure 8), highlighting significant regulatory pairs. Among the identified miRNAs, miR-296-3p emerged as a central regulator, targeting several crucial genes such as *PHOSPHO1* and *FBP2*, which are associated with metabolic and developmental processes essential for antler growth. Additionally, miR-140 and let-7e were found to regulate genes involved in cartilage development and tissue homeostasis. Validation using quantitative real-time PCR (qRT-PCR) demonstrated that the expression patterns of selected genes were consistent with the RNA-Seq data (Figure 9), confirming the reliability of the transcriptomic results.

### 3.4. qRT-PCR Verification of DEGs

Eight representative differentially expressed genes (*PHOSPHO1*, *ACP5*, *TFRC*, *MATN4*, *FBP2*, *NFATC2*, *ABCC4*, and *CNMD*) were selected for qRT-PCR analysis. The expression patterns obtained from qRT-PCR were generally consistent with those derived from RNA-seq (FPKM values), supporting the accuracy of the transcriptomic data (Figure 9). For instance, *PHOSPHO1* was significantly upregulated in the RM layer of the high-yield group, while *TFRC* showed higher expression in the PC layer of the high-yield group compared with the low-yielding group. Conversely, *NFATC2* was more highly expressed in the PC layer of the low-yielding group, suggesting a potential inhibitory role in chondrogenic differentiation.

## 4. Discussion

The antler exhibits one of the fastest growth rates among mammalian tissues, making it a valuable model for studying rapid tissue growth and differentiation. Its unique biological characteristics provide important insights into mechanisms underlying accelerated organ development [11]. By comparing the mRNA and miRNA expression profiles of the D, RM, PC, and C antler layers in high- and low- yield sika deer, this study aims to reveal the molecular mechanisms affecting antler production, and to provide a scientific basis for optimizing antler production and sika deer breeding.

Antlers have long been considered the most economically important trait in sika deer production [28]. Analysis of antler weight revealed significantly higher values in the high-yield group compared with the low-yield group (*p* < 0.01), indicating that genetic factors play a key role in antler growth. Previous research has shown that antler yield is highly heritable [29] and can be effectively enhanced through selective breeding. A previous study conducted whole-genome resequencing of high- and low-yield sika deer populations, identifying 94 genetic variants significantly associated with antler weight. These variants were closely linked to genes involved in antler growth and development [24]. In our study, transcriptomic analysis further elucidated the molecular mechanisms underlying these phenotypic differences, particularly at the mRNA and miRNA expression levels.

During the process of antler growth, the molecular regulatory mechanisms that exist between the various tissue layers are of an extremely complex nature. The cell populations that are present in each layer regulate antler proliferation, differentiation, and maturation through the use of fine molecular signaling and interactions. In our study, we found that there are considerable differences in the expression levels of functional genes in different tissues of antler growth centers, which is similar to previous research results. Subsequently, these differentially expressed genes were subjected to functional annotation and signaling pathway analysis. KEGG enrichment analysis revealed multiple pathways significantly associated with antler growth and development. Among these, the PI3K-Akt signaling pathway is a critical regulator of tissue regeneration and plays a pivotal role in cell proliferation, survival, and migration [30]. A recent study discovered that the PI3K-Akt pathway facilitates the proliferation and migration of antler mesenchymal stem cells (MSCs) through HGF/c-Met signaling [31]. The TGF-β signaling pathway has been demonstrated to play a pivotal role in various physiological processes, including cell differentiation, the formation of the extracellular matrix (ECM), and tissue repair [32]. Zhou et al. (2023) demonstrated that TGF-β1 overexpression can enhance chondrocyte proliferation and ECM synthesis, while inhibiting chondrocyte differentiation by regulating the MAPK signaling pathway [33]. ABC transporters are a family of membrane proteins that utilize the energy derived from ATP hydrolysis to facilitate the movement of a diverse array of substrates across biological membranes. These substrates include lipids, steroids, hormones, ions, and other metabolites [34,35]. Antler growth is a highly dynamic process involving rapid cell proliferation, differentiation, and mineralization. These processes require the transport of large quantities of nutrients and signaling molecules for support [36], such as antler growth, which is hormonally regulated, with testosterone and insulin-like growth factor-1 (IGF-1) playing a key role in this process [37,38]. It has been posited that ABC transporters may provide a more adequate material basis for rapid antler growth by transporting these hormones or their metabolites.

Comparative transcriptomic analysis of the four distinct layers of antler tissue revealed layer-specific molecular mechanisms underlying the differences in antler yield. In the RM layer, high-yield antlers exhibited elevated expression of genes associated with metabolism and proliferation. Notably, *FBP2*, a rate-limiting enzyme in gluconeogenesis, also facilitates glycogen synthesis and has been shown to promote cell proliferation [39,40]. *TPT1* (*TCTP*), a highly conserved protein, was also upregulated and is known to regulate cell proliferation, cell cycle progression, and protein synthesis [41,42,43,44,45]. These results suggest that the RM layer in high-yield antlers possesses greater metabolic and proliferative activity to support rapid tissue expansion. In the PC layer, *NFATC2*, a transcription factor primarily involved in immune regulation and differentiation, was more highly expressed in low-yielding antlers. *NFATC2* negatively regulates chondrogenesis by inhibiting chondrocyte proliferation and differentiation, potentially limiting cartilage formation [46,47]. In contrast, *TFRC*, which facilitates iron uptake and regulates TGF-β/BMP signaling pathways by affecting Smad2/5 phosphorylation, was upregulated in high-yield antlers. Its expression has been shown to promote chondrogenic differentiation by enhancing the expression of key markers such as *Sox9* and *Col2a1* [48,49]. In the C layer, which is responsible for cartilage maturation and mineralization, *PHOSPHO1* was significantly upregulated in high-yield antlers. As an essential phosphatase for initiating bone mineralization during endochondral ossification, *PHOSPHO1* deficiency results in severely impaired skeletal calcification, underscoring its functional importance [50,51]. Lastly, in the D layer, *ZEB1* was found to be elevated in high-yield antlers. *ZEB1* promotes angiogenesis by upregulating VEGFA expression and downregulating miR-206, and has also been implicated in osteogenesis through the regulation of Notch signaling [52,53]. These findings suggest that each antler tissue layer exhibits distinct gene expression patterns, which may contribute to the differences in antler growth and yield.

Several differentially expressed miRNAs have been associated with the rapid growth of antlers. Among these, miR-140 plays a crucial role in cartilage development and homeostasis, inhibiting cartilage degradation by regulating the expression of the *ADAMTS-5* gene [54]. This suggests that miR-140 may be significantly involved in the rapid growth of antler, consistent with the findings reported by Jia et al. [55]. Let-7e, a member of the let-7 family, is also highly expressed in chondrocytes. The let-7 family regulates chondrocyte proliferation by targeting genes such as *Ras*, *Hmga2*, and *Igf2bps*, indicating a potential role in the accelerated cartilage formation observed in deer antlers [56]. In addition, miR-296-3p may promote the rapid growth of velvet antlers by enhancing Wnt/β-catenin signaling through the inhibition of ICAT [57], thereby stimulating chondrocyte proliferation and rapid growth.

In this study, we constructed a putative regulatory network of differentially expressed miRNAs and mRNAs to investigate the molecular mechanisms underlying differences in antler yield. Several potential miRNA–mRNA interactions were identified, including miR-296-3p targeting *PHOSPHO1* and *FBP2*, which may play important roles in antler development. However, we acknowledge that functional validation of these predicted interactions is essential to confirm their biological relevance. Due to limitations in funding and available resources, we were unable to perform follow-up molecular validation experiments, such as luciferase reporter assays, within the scope of this study. Furthermore, our analyses were based on a relatively small sample size (six deer, with three individuals per group), which limits the statistical power and generalizability of our findings. The animals used in this study were not genotyped, which prevented us from directly linking genetic variation to gene expression and phenotypic differences. To address these limitations, future studies should incorporate larger sample sizes, and, ideally, integrate genomic and transcriptomic data from the same individuals. Additionally, incorporating other omics approaches, such as epigenomics, will be valuable for building a more comprehensive understanding of the complex regulatory networks that govern antler growth and yield in sika deer.

## 5. Conclusions

This study provides novel insights into the molecular mechanisms underlying differences in antler yield in sika deer by integrating mRNA and miRNA expression profiles across four distinct tissue layers: dermis (D), reserve mesenchyme (RM), pre-cartilage (PC), and cartilage (C), comparing high-yield and low-yield groups. Differentially expressed genes and miRNAs related to cell proliferation, differentiation, and mineralization were predominantly identified in high-yield individuals. Key genes such as *FBP2*, *TPT1*, *TFRC*, and *PHOSPHO1*, together with miRNAs like miR-140, miR-296-3p, and Let-7e, exhibited tissue layer-specific expression patterns that were closely associated with energy metabolism and cellular proliferation and differentiation. The constructed miRNA–mRNA regulatory networks highlighted potential key regulatory relationships, particularly miR-296-3p–PHOSPHO1 and miR-296-3p–FBP2, which may play central roles in influencing antler growth traits. Additionally, the enrichment of signaling pathways such as PI3K-Akt and TGF-β suggests their involvement in promoting chondrogenesis and ossification. Overall, these findings contribute to a better understanding of the molecular basis of antler growth and yield, and provide a theoretical foundation for selective breeding strategies in the deer farming industry.

## Figures and Tables

**Table 1:** Statistical comparison of deer antler weights between high and low groups.

Items	High-Yield Group (H)	Low-Yield Group (L)
deer antler weights	2.738 ± 0.278	0.782 ± 0.098

*t*-test revealed a significant difference in deer antler weights between the high and low phenotype groups (*p* < 0.01).

**Table 2:** The primer information of qRT-PCR.

Gene Name	Forward Primer (5′→3′)	Reverse Primer (5′→3′)
*PHOSPHO1*	CATCTCAGACGCCAACACCT	GAAGGGTTGCTGAAGATGCG
*ACP5*	GAGCCGGAAGTCACTGTCTC	GAGGCAATCAAGTTCCCCGA
*TFRC*	GCTGCTTTCCCTTTCCTTGC	TCTCCGCCCAGTGTCTCATA
*MATN4*	CAGTGTGTGAGTGAGGGCTT	TCAACGAGCAGAACCAGGTC
*FBP2*	GGACATGCTTACCCTGACCC	TGGAGTTGAGTAGCTGCGTG
*NFATC2*	TGGGCAGCAAATTTGGGAGA	TCCGAATGTGCTTGTTCCGA
*ABCC4*	AGTGAAGGACAGAAAGCCCG	GTTCGAACAAGTGCCTGCTG
*CNMD*	AGCATGACATTCGACCCCAG	CCAGGGGCTCACAGATCTTC
*β-actin*	GCGTGACATCAAGGAGAAGC	GGAAGGACGGCTGGAAGA

## Data Availability

The datasets used or analyzed in the current study are available from the corresponding author upon reasonable request.

## Supplementary Materials

The following supporting information can be downloaded at https://www.mdpi.com/article/10.3390/ani15131964/s1, Table S1: Sequencing data statistics and comparison results. Table S2: Differentially expressed transcripts in the D layer (High-Yield vs. Low-Yield). Table S3: Differentially expressed transcripts in the RM layer (High-Yield vs. Low-Yield). Table S4: Differentially expressed transcripts in the PC layer (High-Yield vs. Low-Yield). Table S5: Differentially expressed transcripts in the C layer (High-Yield vs. Low-Yield). Table S6: Differentially expressed miRNA in the D layer (High-Yield vs. Low-Yield). Table S7: Differentially expressed miRNA in the RM layer (High-Yield vs. Low-Yield). Table S8: Differentially expressed miRNA in the PC layer (High-Yield vs. Low-Yield). Table S9: Differentially expressed miRNA in the C layer (High-Yield vs. Low-Yield). Figure S1: Differentially expressed miRNAs between high-yield (H) and low-yield (L) groups across four tissue layers.
