# Supplementary material for: Integrated mRNA and miRNA Analysis Reveals Layer-Specific Mechanisms of Antler Yield Variation in Sika Deer
Source: Animals (Basel). 2025 Jul 4;15(13):1964. doi: 10.3390/ani15131964 (PMC12248483; doi:10.3390/ani15131964)
Supplement: Supplementary file 1 [file animals-15-01964-s001.zip › Supplementary Figures.pdf]

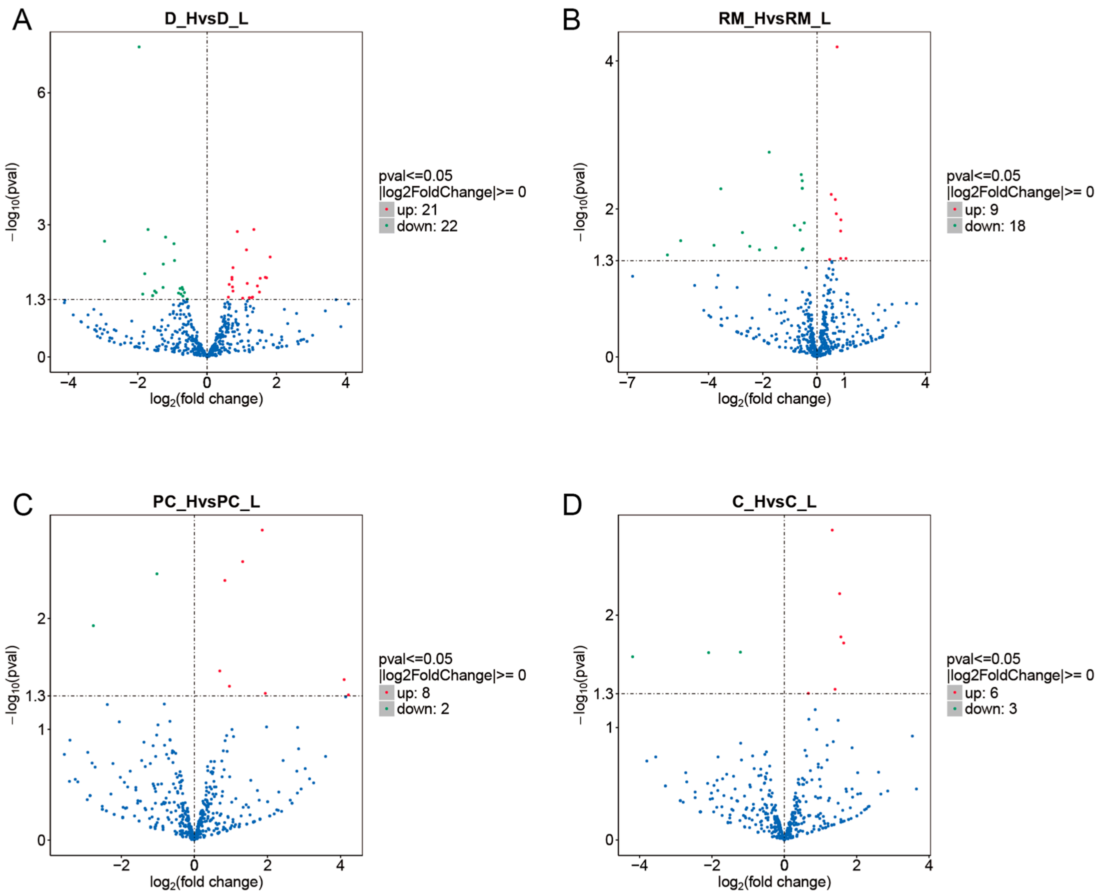

Figure S1. Differentially expressed miRNAs between high-yield (H) and low-yield (L) groups across four tissue layers. Each point represents a miRNA, with the x-axis indicating the log2 fold change (H vs L) and the y-axis indicating the statistical significance (–log10 p-value). Green points represent miRNAs with significantly higher expression in the low-yield group, and red points represent miRNAs with significantly higher expression in the high-yield group (adjusted p-value < 0.05 and |log2FoldChange| > 0). Blue points represent miRNAs with no significant expression difference. (A) Dermis (D); (B) Reserve mesenchyme (RM); (C) Pre-cartilage (PC); (D). Cartilage (C)
